# Vasculitis, Atherosclerosis, and Altered HDL Composition in Heme-Oxygenase-1-Knockout Mice

**DOI:** 10.1155/2012/948203

**Published:** 2012-02-20

**Authors:** Kazunobu Ishikawa, Mohamad Navab, Aldons J. Lusis

**Affiliations:** ^1^The First Department of Internal Medicine and Center for Medical Education and Career Development, Fukushima Medical University, 1 Hikarigaoka, Fukushima 960-1625, Japan; ^2^Division of Cardiology, Department of Medicine, David Geffen School of Medicine at UCLA, Los Angeles, CA 90095-1679, USA

## Abstract

To elucidate roles of heme oxygenase-1 (HO-1) in cardiovascular system, we have analyzed one-year-old HO-1-knockout mice. Homozygous HO-1-knockout mice had severe aortitis and coronary arteritis with mononuclear cellular infiltration and fatty streak formation even on a standard chow diet. Levels of plasma total cholesterol and HDL were similar among the three genotypes. However, homozygous HO-1-knockout mice had lower body weight and plasma triglyceride. HO-1-deficiency resulted in alteration of the composition of HDL. The ratio of apolipoprotein AI to AII in HO-1-knockout mice was reduced about 10-fold as compared to wild-type mice. In addition, paraoxonase, an enzyme against oxidative stress, was reduced less than 50% in HO-1-knockout mice. The knockout mice also exhibited significant elevation of plasma lipid hydroperoxides. This study using aged HO-1-knockout mice strengthened the idea that HO-1 functions to suppress systemic inflammation in artery wall and prevents plasma lipid peroxidation.

## 1. Introduction

 Heme oxygenase (HO) oxidatively catalyzes heme to biliverdin, carbon monoxide, and free iron using NADPH-cytochrome P450 reductase as an electron donor [1–3]. A number of studies suggest a potential protective function of this enzyme against oxidative stress under various conditions with transcriptional activation of heme oxygenase-1 (HO-1) [4, 5]. The antioxidant activity of HO derives from both the elimination of prooxidant heme and the biological activities of its products. Biliverdin and its metabolite by biliverdin reductase, bilirubin, effectively inhibit LDL oxidation [6, 7]. Free iron regulates ferritin synthesis through an iron-responsive element [8]. Ferritin has been shown to have cytoprotective effects against oxidative injuries [9]. Carbon monoxide (CO) modulates the activity of soluble guanylate synthetase like nitric oxide [10] and p38 mitogen-activated kinase activity [11].

 Accumulating evidence suggests that oxidized LDL (oxLDL) plays an important role during the early phases of atherogenesis via its proinflammatory properties [5]. We recently reported that HO-1 is remarkably induced by mildly oxLDL in both endothelial cells and smooth muscle cells [12]. HO-1 expression was also highly responsive to oxidized bioactive oxidized phospholipids existing in LDL [12]. In addition, using artery wall cocultures we found that HO-1 inhibits oxLDL-dependent monocyte chemotaxis through its products bilirubin and biliverdin [12]. We then performed in vivo studies to examine the role of HO-1 on the development of atherosclerosis. HO-1 was highly expressed in atherosclerotic lesion in C57BL/6J, apoE-knockout, and LDL-receptor-knockout mice [13]. When we modulate HO expression in high-fat-fed LDL-receptor knockout mice, HO inhibition significantly resulted in the progression of atherosclerotic lesion formation compared to the mice in which HO was induced [13]. These results lead us to hypothesize the protective roles of HO-1 for atherogenesis.

 High-density lipoproteins (HDLs) are considered to work for antiatherogenesis. These antiatherogenic properties have been explained by reverse cholesterol transport from cells to liver [14]. However, previous studies revealed that HDL itself works as an antioxidant for minimally oxidized low-density lipoproteins [15] and that paraoxonase and apolipoprotein AI in HDL play an important role as antioxidants [16, 17]. However, it is reported that oxidative modification of HDL loses the effect to stimulate efflux of cholesterol from foam cells [18] and that oxidized HDL activates platelets similar to oxidized LDL [19].

 To further strengthen the idea that HO-1 functions as an anti-inflammatory enzyme in artery wall and that HO-1 exhibits antioxidative effects on plasma lipoproteins, we analyzed one-year-old HO-1-knockout mice bred on a standard chow diet. Studies using HO-1 knockout mice [4, 20, 21] revealed that (i) HO-1-homozygous-knockout (HO-1^−/−^) mice develop an anemia with accumulation of iron in liver and kidney, (ii) cultured HO-1^−/−^ embryonic fibroblasts produce high free radicals when exposed to hydrogen peroxides, paraquat, or cadmium chloride, (iii) HO-1^−/−^ mice are vulnerable to mortality when challenged with endotoxin, and (ix) HO-1 expression ensures to survive cardiac xenograft.

 In this study, we examined artery walls and plasma lipoproteins of male HO-1-knockout mice between 1- and 1.5-year old which were bred on a standard chow diet. HO-1-knockout mice had severe infiltration of mononuclear cells at their ascending aortic wall and coronary arteries and small atherosclerotic lesion in aortic sinus. In HO-1 knockout mice, HDL appears to be oxidized. Furthermore, the composition of apolipoprotein AI and AII in HDL altered. These results show HDL oxidation in HO-1-knockout mice. In addition, HO-1-knockout mice showed lower plasma paraoxonase level and higher lipid peroxide level, suggesting that the absence of HO-1 resulted in the oxidation of plasma lipoproteins and activate inflammatory responses in arterial wall.

## 2. Materials and Methods

### 2.1. Reagents

 Reagents utilized were obtained from Sigma unless otherwise specified.

### 2.2. Animal Handling and Procedures

All animal experiments were conducted in accordance with the guidelines of the UCLA and Fukushima Medical University Animal Research Committee. The generation of mice containing targeted disruption of the HO-1 gene was done as previously described [4]. HO-1-deficient mice were generated with C57BL/6J and 129/sv mixed genetic background. C57BL/6J mice for backcross were purchased from the Jackson Laboratory (Bar Harbor, ME). The mice were fed a standard rodent chow diet containing 4% (wt/wt) fat and <0.04% (wt/wt) cholesterol (Oriental Bio, Tsukuba, Japan). Animals were housed four to five per cage and maintained in a temperature-controlled room with a 12-hour light/dark cycle, and animals were strictly monitored for microorganisms.

### 2.3. Atherosclerotic Lesion Analysis

 Following sacrifice, the heart and proximal aorta were excised and washed in phosphate-buffered saline to remove blood. The basal portion of the heart and proximal aorta were embedded in OCT compound (Tissue Tek, Elkhart, IN), frozen on dry ice, and stored at −70°C until they were sectioned. Serial 10-*μ*m cryosections were collected on poly-D-lysine-coated slides, stained with oil red O and hematoxylin, and examined by light microscopy. Atherosclerotic lesion area was calculated using serial sections of the first 400 *μ*m of the ascending aorta as previously described [22]. Hematoxylin-eosin and Elastica Van Gieson staining were done with the paraformaldehyde fixed sections.

### 2.4. Hematocrit, Plasma Lipoprotein Analyses, and Lipid Peroxidation Assay

 Blood was collected from the retro-orbital plexus of mice fasted overnight using heparin-coated capillaries (Fisher Scientific) into a heparin-treated Microtainer tube (Becton Dickinson) and centrifuged at 4°C. The hematocrit was determined by the use of capillary microhematocrit technique in blood obtained. Plasma cholesterol and triglyceride concentrations were determined enzymatically as described previously [23].

### 2.5. Plasma Lipoproteins and Lipid Analyses

 Lipoprotein fractions from pooled mice plasma were isolated by fast performance liquid chromatography [23]. Lipoprotein concentrations are expressed according to their protein content. The characterization of isolated HDL fractions from mouse plasma has previously reported [24]. The protein content of lipoproteins was measured using the method of Lowry et al. [25]. HDL fraction was electrophoresed by 1% agarose gel and stained with Nile Red. SDS-PAGE with 4–20% gradient gel was performed according to the procedure of Laemmli [26].

### 2.6. Paraoxonase Assay

 Paraoxonase activities were assayed using paraoxon as substrate [27]. The cuvette contained 1.0 mM paraoxon in 20 mM Tris/HCl (pH 8.0). The reaction was initiated by the addition of the plasma, and the increase in the absorbance at 405 nm was recorded. Blanks were included to correct for spontaneous hydrolysis of paraoxon. Enzymatic activity was calculated from the molar extinction coefficient 1310 M^−1^ cm^−1^. 1 unit of paraoxonase activity is defined as 1 nmol of 4-nitrophenol formed per min under the above assay conditions [27]. For purified paraoxonase standard solutions, paraoxonase isoforms were isolated as described previously [27].

### 2.7. Other Procedures

Plasma lipid peroxidation as a malondialdehyde was measured with a kit from Oxis (Portland, OR). All values are expressed as means ± SD. Significant difference was determined by one-way ANOVA analysis with Fisher's post hoc test. *P* < 0.05 was considered significant.

## 3. Results and Discussion

### 3.1. Aortitis, Coronary Arteritis, and Atherosclerosis in HO-1-Knockout Mice

**Figure 1:**
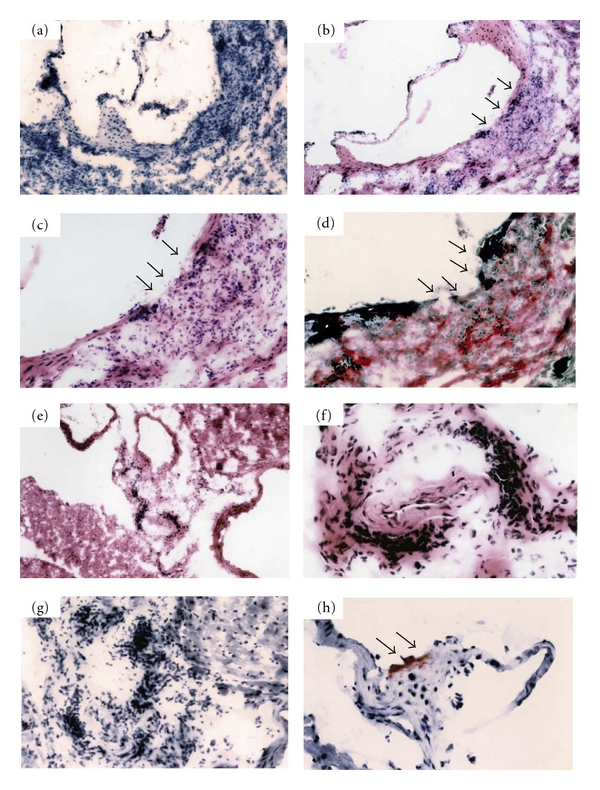
Severe aortitis, coronary arteritis and small atherosclerotic lesion on a standard rodent chow diet after 1 year old of HO-1^−/−^ mice. (a, g, h) Oil red O staining. (b, c, e, f) Hematoxylin- eosin staining. (d) Elastica Van Gieson staining. (d) Elastic fibers in the vessel walls are torn at the site of severe mononuclear cell infiltration. Magnifications at (a, e) ×40, (b) ×100, (c, d) ×200, and (f, g, h) ×400. Predominant sites of aortitis (b, c, d) and initial fatty streak formation (h) are indicated by arrows. These photomicrographs are representative of numerous sections examined.

 To elucidate the effect of HO-1 on vascular system, we examined male HO-1-knockout mice aged over 1 year which were bred on a standard rodent chow diet. These mice had a mixed genetic background of 129Sv and C57BL/6J. HO-1^−/−^ mice had severe infiltration of mononuclear cells at their ascending aortic wall (100%) (Figures 1(a) and 1(b)). This infiltration was transmural, and the intimal elastic laminal structure was severely destroyed (Figures 1(c), and 1(d)). This mononuclear cellular infiltration was not only observed at aorta but also observed at coronary arteries (Figures 1(e), 1(f), and 1(g)). Oil red O staining revealed small atherosclerotic lesion in aortic sinus of HO-1^−/−^ mice even under a standard chow diet though wild-type mice did not develop such lesions (Figure 1(h)). These inflammatory changes of arteries were also observed in heterozygous mice; however, this was less frequently. It is unclear whether these arterial inflammations in HO-1-deficient mice are the response against microorganisms or autoimmune response [28].

### 3.2. Altered HDL Properties in HO-1-Knockout Mice

**Figure 2:**
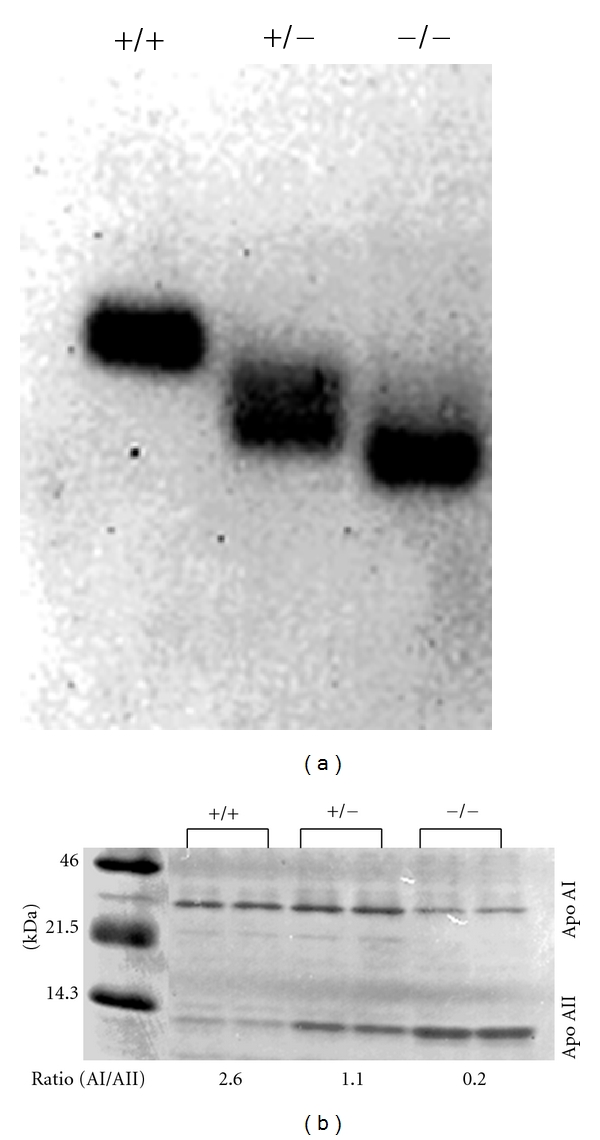
(a) Agarose electrophoresis of HDL in HO-1-knockout mice. 11 *μ*g protein of HDL fraction was electrophoresed in 1% agarose gel and stained with Nile Red. (b) Change of Apolipoprotein AI/AII ratio in HO-1-knockout mice. 1 *μ*g protein HDL was subjected to SDS-PAGE and stained with Nile Red.

Table 1 shows body weights and plasma lipid levels of HO-1-knockout mice analyzed. HO-1^−/−^ mice were significantly lighter than HO-1 wild-type (HO-1^+/+^) and HO-1-heterozygous-knockout (HO-1^+/−^) mice. There were no differences in total cholesterol, HDL, and free fatty acid levels among three genotypes; however, triglyceride levels of HO-1^−/−^ and HO-1^+/−^ were much lower than those of HO-1^+/+^ mice. HDLs were prepared by discontinuous density gradient ultracentrifugation from pooled fresh plasma of each genotype, electrophoresed on 1% agarose gel and stained with Nile Red. 11 *μ*g HDL of HO-1^−/−^ and ^+/−^ mice runs faster than that of HO-1^+/+^ mice, suggesting HDLs of HO-1^−/−^ and ^+/−^ mice are more negatively charged or smaller in size (Figure 2(a)). To examine the composition of apolipoproteins in HDL, 1 *μ*g HDL was subjected to 4–20% SDS-PAGE gel. The ratios of apolipoprotein AI and apolipoprotein AII were different among three genotypes (Figure 2(b)). Apolipoprotein AI was major protein in HO-1^+/+^, whereas apolipoprotein AII was a major in HO-1^−/−^ mice. The ratios of apolipoprotein AI to AII were 2.6, 1.1, and 0.2 in HO-1^+/+^, ^+/−^, and ^−/−^ mice, respectively, by densitometric analyzes. There are more than 10-fold changes of the ratio of apolipoprotein AI to AII between HO-1^+/+^ and HO-1^−/−^ mice. Studies using transgenic mice models suggested that apolipoprotein AI is antiatherogenic [29] and apolipoprotein AII is atherogenic [23]. Though we cannot explain the mechanism why the absence of HO-1 resulted in the compositional change of HDL and produced oxidized HDL, it may be possible to understand HO-1 functions to prevent HDL particle and apolipoproteins from oxidative stress. It is reported that apolipoprotein AI starts denaturation and is easy to make oligomers in oxidized HDL [30]. However, in this study, we did not find oligomeric bands. It will be also interesting to examine whether HO-1^−/−^ mice have more susceptibilities to atherogenesis by high-fat diet challenges.

**Figure 3:**
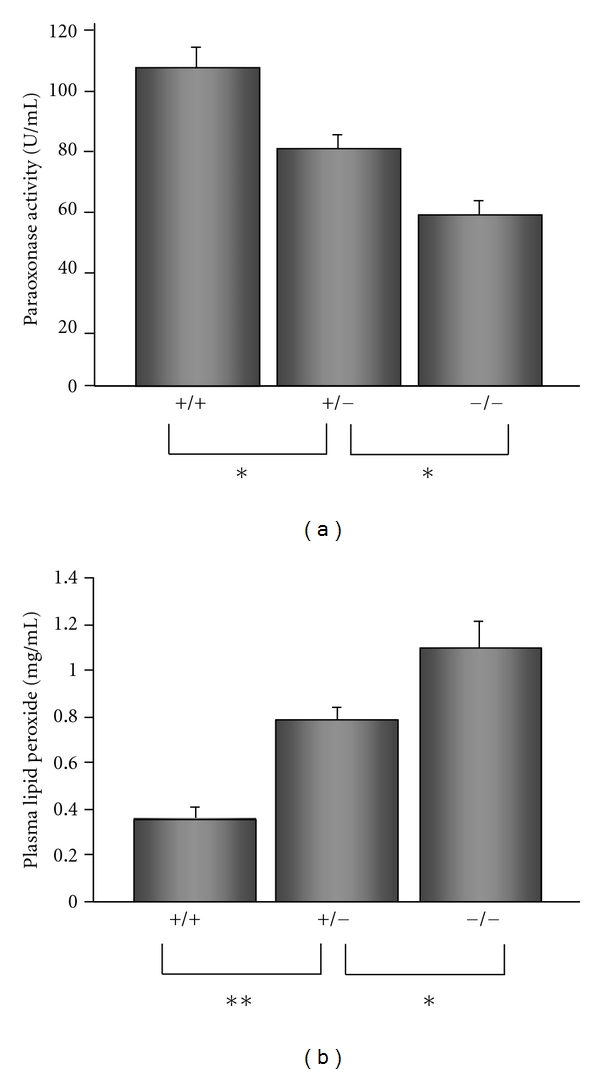
(a) Plasma paraoxonase activities in HO-1-knockout mice. PON activities were determined using arylesterase as substrate and are given as a percentage ± S.D. (b) Plasma lipid hydroperoxide levels in HO-1-knockout mice. Estimates of lipid peroxidation products were obtained by malondialdehyde (MDA) measurements of plasma. Data represents mean ± S.D. from duplicate. Asterisks represent statistically significant differences (*P < 0.05, **P < 0.01).

 Paraoxonase is a calcium-dependent esterase that is known to catalyze hydrolysis of organophosphates and widely expressed in the liver, kidney, intestine, and plasma [31]. Paraoxonase has been suggested to contribute to antioxidant protection of HDL to LDL oxidation in vitro [32] and in vivo [33]. HO-1^−/−^ mice had decreased plasma paraoxonase activity less than 50% compared to HO-1^+/+^ mice (Figure 3(a)). This reduced paraoxonase activity may imply that HDL in HO-1^−/−^ mice was suffered by stronger oxidative stress. There may be unknown relationship between reduced paraoxonase activity and the compositional changes of apolipoprotein AI to AII. Plasma lipid peroxides levels of HO-1^−/−^ mice were higher than those of HO-1^+/+^ mice (Figure 3(b)), suggesting that HO-1 functions to suppress lipoprotein oxidation presumably by production of antioxidants, biliverdin and bilirubin.

 Over 95% of HO-1^−/−^ mice die in utero with unknown reason [4]. It may be not easy for HO-1^−/−^ mice to survive without intrinsic antioxidant systems although the reason only a part of those survives is not still clear. In addition, changes in HO-1^−/−^ mice such as lower body weight and decrease of plasma triglyceride need to be further examined by analyzing food consumption and metabolic rate. However, our data using HO-1^−/−^ mice directly suggests a significant function of HO-1 as an anti-inflammatory molecule in artery wall and for native HDL.

## Figures and Tables

**Table 1:** Body weight and plasma lipid levels of HO-1-knockout mice.

HO-1 genotype	+/+(*n* = 7)	+/−(*n* = 6)	−/−(*n* = 5)
Body weight (g)	45.6 ± 4.3	43.8 ± 3.8	30.6 ± 3.9*
Hematocrit (%)	46 ± 4	44 ± 2	41 ± 2
Total cholesterol (mg/dL)	92 ± 18	87 ± 11	86 ± 15
Triglyceride (mg/dL)	106 ± 38	26 ± 4	14 ± 3*
HDL-cholesterol (mg/dL)	73 ± 8	72 ± 11	65 ± 9
Free Fatty Acid (mg/dL)	43 ± 7	41 ± 8	36 ± 3

Lipid levels are given in mg/dL ± S.D. Values for lipid levels on a standard chow diet were from mice of 50% C57BL/6 and 50% 129/Sv genetic background. *P < 0.05.

## Abbreviations

HO: Heme oxygenase
HDL: High-density lipoprotein
LDL: Low-density lipoprotein
oxLDL: Oxidized LDL
−/−: Homozygous knockout
+/−: Heterozygous knockout
+/+: Wild type.
